# Cocaine-Induced Acute Pancreatitis: A Rare Etiology

**DOI:** 10.7759/cureus.9029

**Published:** 2020-07-06

**Authors:** Muhammad Umar, Erum Noor, Unaiza Ali, Israr Khan, Zahoor Ahmed

**Affiliations:** 1 Internal Medicine, Jinnah Sindh Medical University, Karachi, PAK; 2 Internal Medicine, Jinnah Medical and Dental College, Karachi, PAK; 3 Internal Medicine, Ziauddin University, Karachi, PAK; 4 Internal Medicine, Bolan Medical College, Quetta, PAK; 5 Internal Medicine, King Edward Medical University, Mayo Hospital, Lahore, PAK

**Keywords:** cocaine, acute pancreatitis, management

## Abstract

An 18-year-old male with a medical history of trigeminal neuralgia presented to the emergency department with complaints of severe abdominal pain associated with nausea, projectile vomiting, and watery diarrhea with no fever, rigors, and chills. The abdominal examination was unremarkable. His lab results showed elevated serum lipase and amylase. Gallstones were ruled out by abdominal ultrasonography. His computed tomography (CT) revealed pancreatic enlargement with ill-defined borders. He reported cocaine use but had no history of alcohol abuse. A urine drug screen was positive for cocaine. He was managed conservatively with a possible diagnosis of acute pancreatitis due to cocaine abuse after carefully ruling out other causes. The patient was symptom-free on day 7 and discharged from hospital on day 8 with follow-up with his gastroenterology doctor and drug counseling service. Although cocaine-induced pancreatitis is rare, it should be considered a differential diagnosis in patients with a history of cocaine use.

## Introduction

Pancreatitis is a common gastrointestinal (GI) disease with a diverse spectrum of presentation based on its severity. Acute pancreatitis (AP) is clinically characterized by either mild, self-limiting, or severe disease with dangerous complications. Its annual incidence rate has increased globally, ranging from 13 to 45 cases per 100,000 persons on average [1]. There are many causes of AP but gallstone and binge alcohol consumption are considered to be the most common etiologies of AP [2,3]. Drug-induced pancreatitis is among the less common causes [4,5], and in this subgroup, cocaine-induced pancreatitis is uncommon [6,7]. Here, we present a rare case of AP in a young patient caused by cocaine ingestion.

## Case presentation

An 18-year-old male with a medical history of trigeminal neuralgia presented to the emergency department with complaints of severe epigastric pain. The pain was dull, started suddenly, and worsened gradually. The patient reported that he had this pain for the last 12 hours associated with nausea and three episodes of projectile vomiting containing food particles with no fever, rigors, and chills. He denied alcohol abuse and had no family history of any malignancy. However, the patient admitted having cocaine abuse for the last one week. The initial evaluation revealed a temperature of 98°F, blood pressure of 100/70 mmHg, respiratory rate of 22 breaths/minute, and heart rate of 102 beats/minute. The abdominal examination revealed normal bowel sounds with no distension.

Initial laboratory analysis is given in Table 1 and Table 2. His serum metabolic panel was unremarkable except for elevated serum lipase and amylase and mild elevation of serum creatinine and total bilirubin.

**Table 1:** Results of hematological examination

Parameter	Admission value	Normal range
White blood cell count, cells/mm^3^	9000	4000-11000
Red blood cell count, million cells/mm^3^	4.5	4.35-5.65
Hemoglobin, g/dL	14.1	14-17
Hematocrit (%)	43.9	41-51
Platelet count/mm^3^	300,000	150,000-350,000

**Table 2:** Comprehensive metabolic panel ESR, erythrocyte sedimentation rate

Parameter	Admission value	Normal range
Lipase (IU/L)	2201	0-160
Amylase (IU/L)	602	30-110
Alkaline phosphatase (mg/dL)	71	36-92
Aspartate aminotransferase (IU/L)	40	8-­48
Alanine aminotransferase (IU/L)	35	7-55
Prothrombin time (second)	12.4	11-13.5
Partial thromboplastin time (seconds)	29	30-40
Total bilirubin (mg/dL)	2.3	0.3-1.2
C-reactive protein (mg/dL)	15.3	<10
ESR	19	0-22
Sodium (mmol/L)	141	136-145
Potassium (mmol/L)	3.9	3.5-5.0
Chloride (mmol/L)	100	98-106
CO_2_ (mmol/L)	27	23-38
Urea nitrogen (mg/dL)	14	8-20
Creatinine (mg/dL)	1.5	07-1.2
Blood glucose (mg/dL)	85	70-100 (fasting)
Total protein (mg/dL)	5.9	6.0-7.8
Albumin (mg/dL)	4.1	3.5-5.5
Calcium (mg/dL)	9.6	9.0-10.5

Abdominal ultrasound revealed normal-sized liver and biliary ducts with no evidence of gallstone or biliary stone. A lipid panel was performed, and his LDL (low-density lipoprotein cholesterol) was 43 mg/dL, HDL (high-density lipoprotein cholesterol) was 55 mg/dL, serum cholesterol was 135 mg/dL, and serum triglyceride was 128 mg/dL. His urine drug screening confirmed cocaine abuse. He was admitted to the medical intensive care unit with a possible diagnosis of AP due to cocaine use as the patient had no other risk factor for AP.

**Figure 1:**
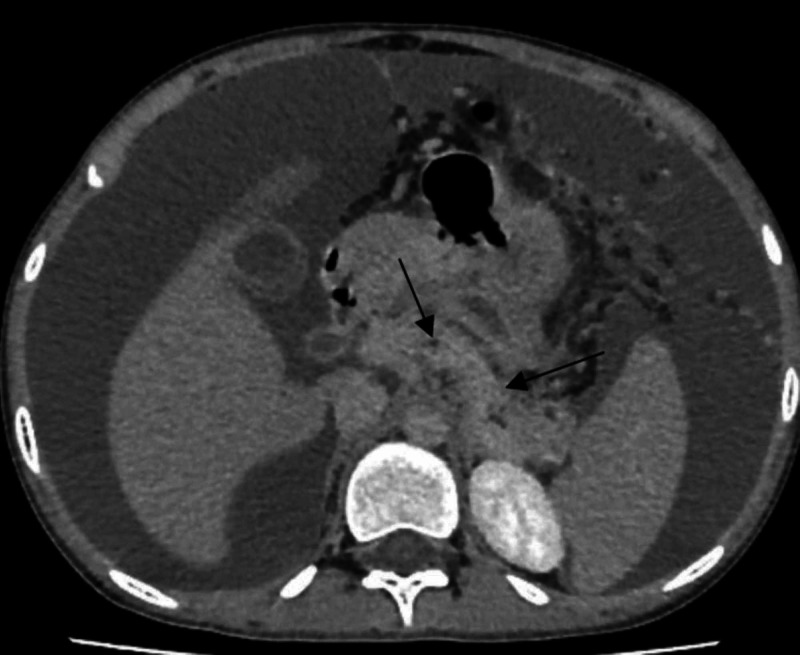
CT showing pancreatic enlargement with ill-defined border

**Figure 2:**
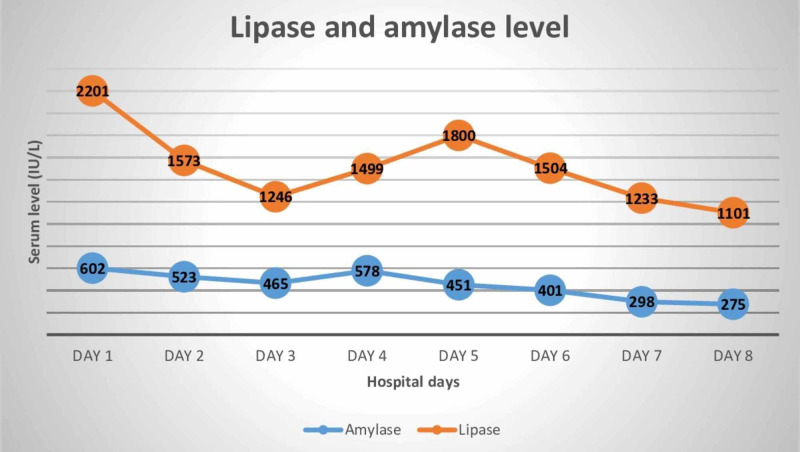
Daily levels of amylase and lipase

The patient was managed conservatively. The patient was kept NPO (nil per os) and was resuscitated with isotonic fluids and analgesia. On the second hospital day, improvement in the patient’s clinical condition was observed and enteral nutrition was initiated. However, the pain did not improve. His pancreatic enzymes remained elevated. The patient was switched to parenteral nutrition again due to oral feed intolerance and pain in the epigastric region radiating to the back. His computed tomography (CT) revealed pancreatic enlargement with an ill-defined border (Figure 1). Daily levels of lipase and amylase were analyzed (Figure 2). The patient was symptom-free at hospital day 7 and was started on oral nutrition again. On day 8, he was discharged from the hospital with follow-up with his gastroenterology doctor as well as drug counseling service.

## Discussion

Cocaine (benzoylmethylecgonine), a crystalline tropane alkaloid, is extracted from the leaves of the Erythroxylum coca plant [8]. Cocaine is a drug of abuse and has four main routes of abuse: ingestion, smoking, inhalation, and injecting. However, the basic patent compound remains the same and has a parallel metabolic profile picture irrespective of the form. The effects of cocaine differ from the route of administration. Cocaine has multisystem effects involving GI, neurologic, psychiatric, obstetric, and cardiopulmonary systems. Cardiovascular and neurologic manifestations are prime alterations [9]. GI manifestations of cocaine abuse are infrequent and usually present with bowel ischemia, bloody stool, and bowel perforation. However, isolated pancreatic involvement is rarely reported [6].

Cocaine has a wide range of presentation based on its potential inhibitory action on dopamine, norepinephrine, and serotonin reuptake. Additionally, it acts as a central nervous system stimulant. Short-term clinical features include vasoconstriction, high blood pressure, increase heart rate and energy, hypervigilance, pupillary dilation, change in appetite, and temperature surge. Moreover, it also predisposes to thrombus formation secondary to platelets aggregation through direct vasoconstrictive effect on endothelial cells [8]. The bowel complications of cocaine abuse are a direct consequence of the negative effect of cocaine on neurotransmitter reuptake by presynaptic neurons, which result in the increased local concentration of neurotransmitters at the site of the neurotransmitter receptors causing ischemia, inflammation, and ulcer of the bowel, which result in abdominal pain, nausea, vomiting, diarrhea, and blood in the stool [10].

Diagnosis of AP is clinical and serological, as well as by imaging modalities. Two of the following three criteria must be met to diagnose AP [11,12]:

1. Upper abdominal pain consistent with the disease activity (e.g., acute onset, epigastric, and usually radiating to the back).

2. Serum lipase and/or amylase level >3x the upper limit of normal.

3. Characteristic AP findings on imaging modalities (such as abdominal CT, MRI, or ultrasonography).

AP is usually managed with supportive care (resuscitation with fluids and analgesia) and nutritional support (enteral nutrition if the patient cannot tolerate an oral diet). The use of prophylactic antibiotics is usually not recommended in AP [13].

In our patient, alcoholic etiology was ruled out because there was no reported history of alcohol abuse. Similarly, no evidence of gallstone was revealed by ultrasound. Furthermore, hypertriglyceridemia as a possible trigger was not considered may be due to the serum triglycerides level of 128 mg/dL shown on the lipid panel. A urine drug screen was positive for cocaine abuse. His improvement with conservative management and lowering of pancreatic enzymes confirmed AP attributable to cocaine abuse.

## Conclusions

Cocaine abuse is a pressing concern due to the rise in the illegal use of cocaine worldwide. Cocaine-induced AP is a rare cause. Other common causes must be ruled out thoroughly; however, it has a complex presentation and can be lethal if not identified and addressed appropriately. Therefore, clinicians should have sound knowledge and pay close attention to crack cocaine use and related GI complications.
